# Implementation and evaluation of a protocol management system for automated review of CT protocols

**DOI:** 10.1120/jacmp.v17i5.6164

**Published:** 2016-09-08

**Authors:** Joshua Grimes, Shuai Leng, Yi Zhang, Thomas Vrieze, Cynthia McCollough

**Affiliations:** ^1^ Department of Radiology Mayo Clinic Rochester Mn USA

**Keywords:** computed tomography, protocol monitoring, protocol management, radiation safety

## Abstract

Protocol review is important to decrease the risk of patient injury and increase the consistency of CT image quality. A large volume of CT protocols makes manual review labor‐intensive, error‐prone, and costly. To address these challenges, we have developed a software system for automatically managing and monitoring CT protocols on a frequent basis. This article describes our experiences in the implementation and evaluation of this protocol monitoring system. In particular, we discuss various strategies for addressing each of the steps in our protocol‐monitoring workflow, which are: maintaining an accurate set of master protocols, retrieving protocols from the scanners, comparing scanner protocols to master protocols, reviewing flagged differences between the scanner and master protocols, and updating the scanner and/or master protocols. In our initial evaluation focusing only on abdomen and pelvis protocols, we detected 309 modified protocols in a 24‐week trial period. About one‐quarter of these modified protocols were determined to contain inappropriate (i.e., erroneous) protocol parameter modifications that needed to be corrected on the scanner. The most frequently affected parameter was the series description, which was inappropriately modified 47 times. Two inappropriate modifications were made to the tube current, which is particularly important to flag as this parameter impacts both radiation dose and image quality. The CT protocol changes detected in this work provide strong motivation for the use of an automated CT protocol quality control system to ensure protocol accuracy and consistency.

PACS number(s): 87.57.Q‐

## I. INTRODUCTION

It is common for CT practices to manage a large number of scanning protocols. For large or enterprise practices, the number of CT protocols can be in the hundreds. Each of these protocols contains dozens of technical parameters appropriately adjusted for specific diagnostic tasks and specific scanner models. With ongoing protocol optimization efforts, large numbers of protocols, and the operation of multiple scanners, inappropriate or inaccurate modifications of protocols can easily occur. For example, this could be caused by typographical errors by the users, who manually enter or change scanning or reconstruction parameters on the scanner, or by inadvertent saving of a patient‐specific protocol into the default protocol. Systematic errors in CT protocols may lead to unnecessary levels of radiation, and in extreme cases, can even lead to radiation injury.^(1)^ At the other extreme, errors in CT protocol parameters may result in suboptimal image quality, jeopardizing a proper clinical diagnosis. To ensure that patients are scanned correctly, all protocols on all scanners should be routinely reviewed for accuracy.^(2)^

Recognizing this need, the American College of Radiology (ACR) and the Joint Commission have made protocol review mandatory for CT accreditation to be sure that no unintended changes have been applied that may degrade image quality or unreasonably increase dose.^(3)^ In addition, some states such as California and Texas now require such protocol reviews.^(2)^ Protocol review involves reviewing of the content of the protocol, and ensuring the right content is programmed on the scanner. The former could be potentially conducted offline if a protocol system (or protocol book) is available, either in hard copy or electronic format. The latter needs to be checked on each individual scanner. However, performing a thorough review is not a small task. There can easily be hundreds of protocols loaded onto a single scanner.^(4)^ Continual protocol customizations and optimizations, and the potential for erroneous or unauthorized changes, require that protocols, as loaded on the scanner, be reviewed frequently. Furthermore, differences between CT scanner manufacturers and between models of scanners from the same manufacturer produce additional challenges to maintaining a consistent set of protocols across a large practice.^(5)^ Considering these factors, manual review is labor‐intensive, error‐prone, and costly. Therefore, an automated approach using computer software is essential.^(6)^

Our objective in this work was to create a protocol management system that could be used to ensure that protocols programmed on the scanners at our large institution were set appropriately, and to periodically monitor these protocols in order to identify and fix inappropriate modifications. This article describes our experience with the development, implementation, and evaluation of this protocol monitoring software. First, we outline the general steps in the protocol‐monitoring workflow. Next, we describe the methods and results of a pilot study performed with the first version of our software. Finally, we summarize updates made to the software based on results from the pilot study.

## PROTOCOL MONITORING WORKFLOW

Our protocol‐monitoring system was developed to ensure that the CT protocols programmed on the scanners matched the set of master protocols designed by a protocol review team. We define a set of master protocols as the institutional set of protocols, such that each individual master protocol has a corresponding protocol programmed on the scanners of the institution (i.e., one master protocol for each clinical protocol). This definition of master protocol differs from the use of this term by Szcykutowicz and Siegelman,^(6)^ who define a master protocol as the set of technical acquisition parameters that give the same level of image quality and can be used for multiple clinical indications.

Maintaining the set of master protocols is a key component of protocol management, which is a complicated process carried out by a protocol review team consisting of at least one radiologist, one medical physicist, and one technologist. Each member of this protocol review team has specific roles and responsibilities for the review, implementation, and verification of protocols used within the institution.^(2)^ A CT protocol refers to the complete set of parameters used to perform a CT examination, including acquisition parameters, patient instructions, contrast administration instructions, and reconstruction parameters.^(7)^ Protocol review refers to the evaluation of all parameters that constitute each CT protocol. This evaluation should be performed periodically in order to optimize protocols and implement new technologies that can be used to improve image quality or reduce patient dose. Another important responsibility of the protocol review team is to verify that the protocol parameters are appropriately programmed onto the scanner and appropriately listed in the master protocol set. This verification step should be part of a regular review process to ensure that no unintended changes have been applied to the master or scanner protocols during manual data entry or by unauthorized editing of the scanner protocols. We refer to these unintended changes as inappropriate changes; that is, the change in parameter was not planned by the protocol review committee. Inappropriate changes can occur on either the scanner, the master protocol, or in both places.

**Figure 1:**
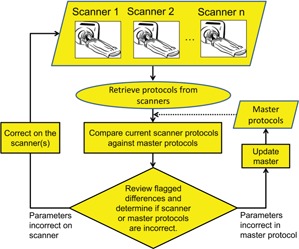
Protocol monitoring workflow.

In this work, we have focused on the verification step of the protocol management process. Specifically, we have designed a protocol monitoring system to verify the technical parameters programmed on the scanner, namely the acquisition and reconstruction parameters. As illustrated in Fig. 1, the key steps in our protocol monitoring workflow for protocol verification are to

- generate and maintain an accurate set of master protocols;
- retrieve protocols from the scanners to the workstation running the protocol monitoring software;
- compare scanner protocols to master protocols;
- review flagged differences between the scanner and master protocols; and
- update the scanner and/or master protocols.

The protocol monitoring tool that we have developed to complete each of these steps was programmed using MATLAB (Mathworks Inc., Natick, MA) and Excel (Microsoft Corporation, Redmond, WA). The development of this software has provided experience with different solutions to each step in the workflow. Further details and considerations for each of these key steps are described below.

### A. Master protocols

Some institutions keep hard copies of protocol instructions in binders in the CT scanner control room, while others have an online set of protocols. Neither guarantees that the protocols are correct on the scanner. For an automated protocol monitoring solution, it is necessary to have an electronic version of the master protocols. There are several options for creating a master protocol list in an electronic format:

1. Protocol parameters can be manually entered into a spreadsheet, text document or database.
2. Protocol files can be exported directly off of the scanner.
3. Protocol parameters can be retrieved from the DICOM header of previous patient exams.

Choosing a suitable option requires consideration of the different benefits and limitations associated with each of these methods. The first option is tedious and manual data entry is prone to error. The second and third options help to automate the process, but assume that the protocols are correctly programmed on the scanner at baseline. The third option may be limited since some parameters may not be included in the DICOM header of patient images. Furthermore, the third option can be problematic because technologists may adjust the default imaging parameters for patient‐ or exam‐specific reasons (e.g., body habitus). Regardless of the method chosen, the generated master protocols require thorough review to ensure that each parameter is set appropriately. Given the importance of protocol management, protocol management solutions have been actively explored by the CT community.^2^, ^6^, ^8^, ^9^ In addition, commercial vendors are also exploring software solutions for protocol management (e.g., Toshiba released a protocol management system in 2015).

Master protocols can be created as one master protocol per protocol per scanner. They can also be created as one master protocol per protocol per scanner model (i.e., scanners with the same vendor and model share the same master protocol for a given protocol). Both of these options will be discussed more in two separate sections below (Pilot Study and Protocol Monitoring System, version 2).

### B. Retrieving protocols from the scanner

Scanner protocol files need to be retrieved from each scanner so that they can be accessed from a central workstation and compared to the master protocols. One option is to copy the protocols from the scanners onto a USB flash drive. This option requires someone physically going to each scanner to export the protocols, which can become tedious depending on the number of scanners and the frequency that the software is going to be run. A second and more efficient option is to automatically retrieve the scanner protocols via the network. An institution may need to work with the scanner manufacturer to find a solution for this second option. Another potential option is to remotely access and transfer CT protocols from scanners to workstations. DICOM is currently working on a standard for CT protocols, which would allow this option to become feasible in the future (S. Leng, personal communication with committee members of this new DICOM standard, supplement 122, September 09, 2015).

### C. Comparing scanner protocols to master protocols

To begin an automated comparison, the software will first need to match each scanner protocol to a corresponding master protocol. Ideally, a protocol labeling system is put in place where each protocol in the master protocol list has been assigned a unique identifier (such as a unique protocol number). These unique identifiers can then be incorporated into the names of the saved scanner protocols. Otherwise, the software will need to rely on matching protocol names, in which case it is more likely for naming variations to cause issues when looking for corresponding protocols.^(10)^ Even differences in spaces or capitalization can potentially cause the matching process to fail. It could be challenging to compare protocol names from different vendors, especially with vendor specific naming conventions. Standardizing and unifying naming conventions is important to any clinical practice, especially to those with equipment from multiple vendors. Scientific societies such as RSNA and AAPM have devoted substantial effort to providing mappings of manufacturer‐specific terminology to more general terminology. These efforts include the RadLex from RSNA and CT Lexicon from AAPM.^7^, ^11^ These can help to standardize the protocol parameter names and series naming conventions. The tool proposed in this study will enable automatic checking of protocol and series descriptions to keep the names consistent.

Another consideration is deciding what protocol parameters to monitor. A typical CT protocol contains a lot of information and many different parameters. Tracking all parameters increases the time required for the manual process of reviewing the output from the software. Currently we monitor the parameters listed in Table 1. While complete protocol documentation might include patient preparation instructions, contrast parameters, billing codes, and other details, we have focused on monitoring the technical components of the protocol, namely the acquisition and reconstruction parameters that are most clinically relevant and that are user‐adjustable on the acquisition console. However, our methods can be easily translated to other protocol information and the system is designed to be flexible in terms of adding or deleting parameters to be monitored.

**Figure 2:**
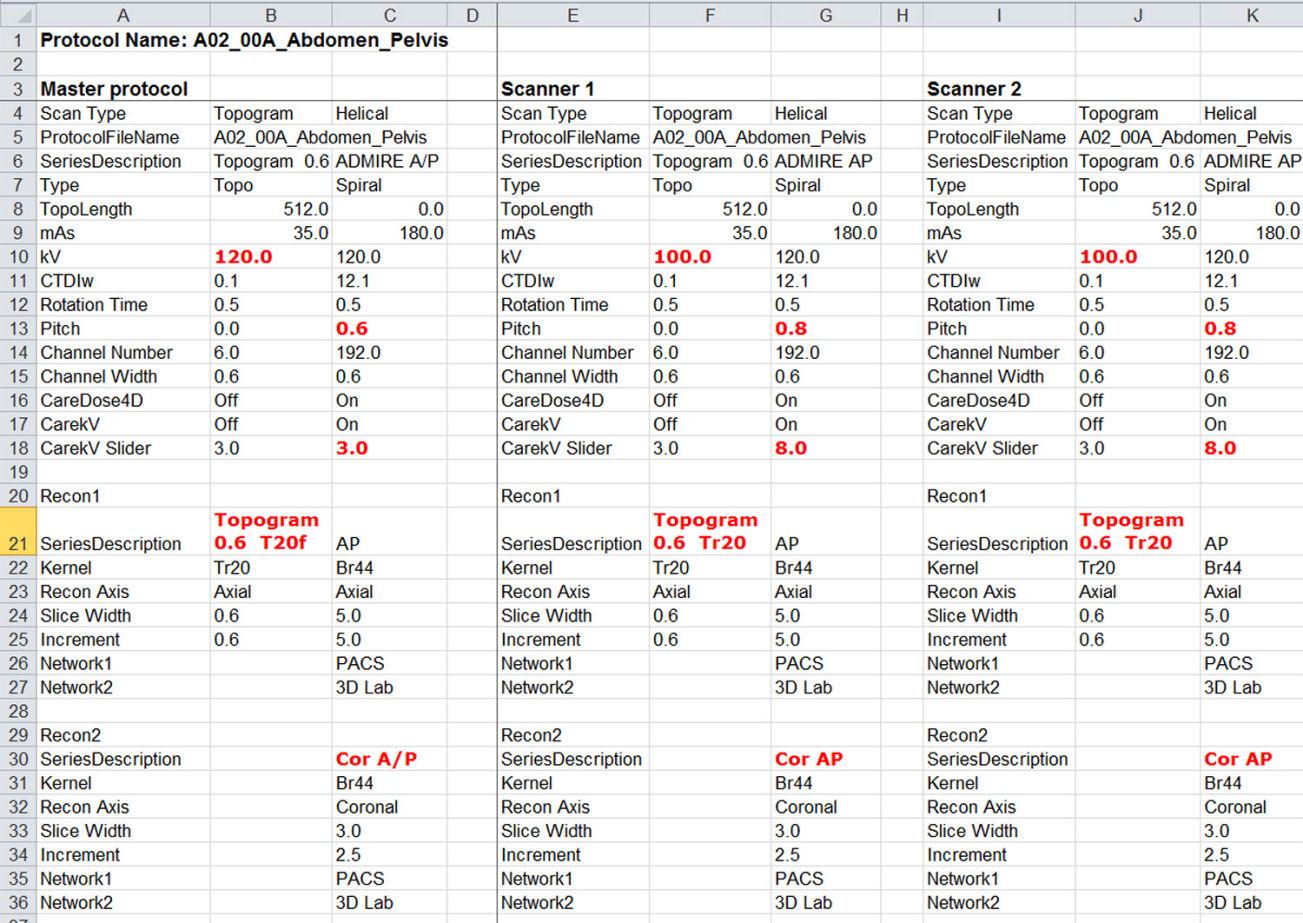
Example results from protocol view showing flagged discrepancies between the master protocol and the protocols programmed on two scanners in series description, helical pitch, and CARE kV slider value.

Finally, detected differences between the scanner and master protocols need to be displayed so that they can be reviewed by appropriate personnel in the next step. In our implementation, a software routine written using the MATLAB programming language and environment was used for reading protocol parameters from the master and scanner protocol files, comparing these parameters, and then displaying the results in a spreadsheet using color‐coding to flag any discrepancies between the master and scanner protocols (Fig. 2).

**Table 1:** Protocol parameters currently being monitored by the software.

*Acquisition*	*Reconstruction*
Scan type (axial or helical)	Series description
Tube current	Kernel
Tube potential	Slice thickness
Pitch	Slice increment
Automatic exposure control settings	Reconstruction direction
Tube rotation time	Network destinations
Collimation	

### D. Reviewing flagged discrepancies

After the scanner and master protocols have been compared, a decision is needed to determine whether any flagged differences are because of a mistake in the parameter setting on the scanner (e.g., due to human error) or in the master protocol. Although it may be possible to automate this decision process in some scenarios, most cases still require a human decision by a member of the protocol review team. We built a user interface in Excel Visual Basic (Microsoft) that allows a reviewer to interact and decide if flagged differences correspond to mistakes in the master or scanner protocol. After all flagged differences are reviewed, this interface produces a summary of the reviewer's decisions for which protocols (master or scanner) are correct. Ultimately, input from the entire protocol review team may be needed to decide which parameters are correct. For example, a physicist may be the best resource to decide which rotation time/AEC setting combination is correct, while a technologist may need to resolve discrepancies with scan direction. If the parameter is wrong on the scanner, then the protocol parameter will need to be updated directly on the scanner. It is also possible that the value on the scanner is correct and the master protocol is wrong, in which case the master protocol will need to be updated. This scenario could arise during a protocol optimization phase where the protocol change on the scanner is made on the fly before adjusting the master protocol. Good practice would be to always make changes in the master protocol first. However, this might not always be feasible in real practice. Therefore, in our implementation, we choose to allow the reviewer to decide what is correct instead of making the assumption that the master protocol is always correct.

It should be emphasized that in the current work the protocol monitoring tool is not being used to review protocols in their entirety. The tool is only being used to review parameters with detected differences between the scanner and master protocols. When reviewing the flagged differences, the reviewer is using the protocol review team's knowledge of how the protocol was designed to decide which version is correct, the master or the scanner.

### E. Updating the scanner and/or master protocol

In the last stage, any flagged differences will require updates to either the scanner or master protocols so that they can be made equivalent. For updating scanner protocols, there is not yet an existing method for automatically updating protocols on multiple scanners simultaneously, which makes it necessary to go to each scanner where there is a discrepancy and update the scanner manually. Once the DICOM standard for CT protocols is released, it should be possible to update protocols on scanners remotely and/or automatically. For updating master protocols, a message can be sent to the protocol review team with a notification that master protocol edits are necessary. Once the changes are approved, the master protocol will be updated by an assigned member of the protocol review team and the changes along with any comments can be documented.

### F. Frequency of protocol monitoring

Another consideration for implementing a protocol monitoring program is to decide how frequently to run the software. If the software runs automatically on a fixed schedule (e.g., daily or weekly), then action is only required when a discrepancy between the scanner and the master protocols is detected, which could trigger an alert sent to the protocol review team. Other times when it may be useful to run the application include immediately following protocol optimizations, after any updates to the master protocols, or any time new protocols are loaded onto a scanner, such as after a software upgrade.^(9)^ This helps to ensure that parameters on all scanners have been accurately entered.

## PILOT STUDY

### A. Methods

We performed a pilot study to evaluate an early version of our CT protocol monitoring system. This study did not require IRB review as it did not involve human subjects. This evaluation focused on abdomen and pelvis protocols on two Siemens Definition Flash scanners (Siemens Healthcare, Forchheim, Germany). Monitored protocols included both single‐ and dual‐energy protocols, as well as adult and pediatric protocols. Protocol monitoring was performed once per week for 24 weeks. Each week, the scanner protocols were exported to a server where the protocol monitoring software could access them. During this pilot study, we tracked all protocol changes on the two scanners. Specifically, the software kept track of how many protocols were deleted, added, and modified each week. The flagged modified protocols were reviewed by lead technologists to determine whether the protocol changes were appropriate or inappropriate modifications, based on the appropriate protocol parameters that had been agreed on by the entire protocol review team, of which the lead technologists are members. Appropriate modifications were defined as intentional protocol changes made correctly on the scanner. These modifications were flagged when the software detected a difference in parameters since the last time the program was run. Inappropriate modifications were defined as changes that were inadvertently saved to the scanner protocol.

In this early version of the protocol monitoring software, the master protocols were based on the protocols loaded on the scanners at Week 1. In subsequent weeks, the scanner protocols were compared to the master protocols, which might have evolved over time. During review of flagged protocol differences, inappropriate scanner protocol modifications were corrected by changing the protocols on the scanner. If a scanner protocol modification was found to be appropriate, then the scanner protocol became the new master protocol.

### B. Results

**Figure 3:**
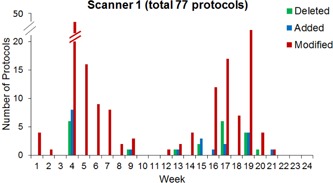
Scanner 1 protocol changes over time, including protocol deletions, additions, and modifications.

**Figure 4:**
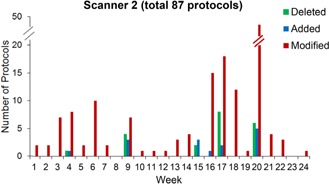
Scanner 2 protocol changes over time, including protocol deletions, additions, and modifications.

The total number of protocols on scanners 1 and 2 varied as protocols were added and deleted over the 24‐week trial (3, 4). During this time period, 309 protocols were modified with a total of 712 protocol parameter modifications on scanners 1 and 2 combined. Broken down by scanner, there was an average of 77 separate abdomen and pelvis protocols present on scanner 1 over the course of the pilot study. These scanner 1 protocols were modified a total of 159 times, with the number of protocols being modified per week varying from no changes up to 46 modified protocols in Week 4 (Fig. 3). This peak in protocol modifications followed a software upgrade on the scanner between Weeks 3 and 4. The reason for these flagged modifications was not due to protocol inconsistencies caused by the software upgrade. Instead, the software upgrade prompted the lead technologists to carefully review the protocols, which led to the discovery of pre‐existing errors in the protocol parameters. Scanner 2 had an average of 87 abdomen and pelvis protocols, which were modified a total of 150 times over the 24‐week period. Similar to scanner 1, peaks in the number of modifications corresponded to periods of protocol review and optimization (Fig. 4).

**Figure 5:**
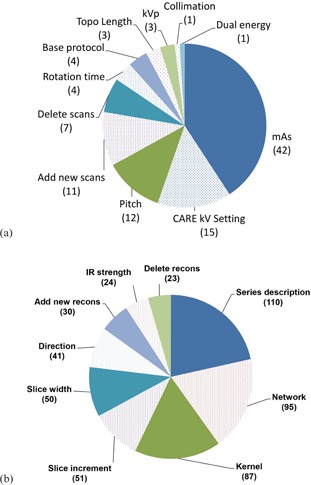
Total number of appropriate modifications to acquisition (a) and reconstruction (b) parameters on scanners 1 and 2. In (a), the CARE kV Setting was either “On” or “Off”, while the kVp referred to the actual tube potential setting.

Review of the protocol modifications found 614/712 (86%) to be appropriate modifications. The majority of these appropriate modifications were changes to the reconstruction parameters. In total, 103/614 (17%) appropriate modifications were acquisition parameter modifications, while 511/614 (83%) appropriate modifications were reconstruction parameter modifications (Fig. 5). The most common appropriate acquisition parameter changes were modifications to the quality reference mAs (effective mAs for a reference size patient), CarekV setting (on or off), and pitch, which were modified a total of 42, 15, and 12 times, respectively (Fig. 5(a)). These results are a reflection of a continual effort to optimize CT protocols and reduce radiation exposure in our practice. The most common appropriate reconstruction parameter changes were modifications to the series description, network destination, and reconstruction kernel, which were modified a total of 110, 95, and 87 times, respectively (Fig. 5(b)).

**Figure 6:**
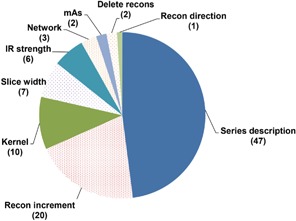
Total number of inappropriate modifications to acquisition and reconstruction parameters on scanners 1 and 2 combined.

Inappropriate modifications were identified in 98/712 (14%) protocol parameter changes. With an average of more than one protocol parameter change per modified protocol, 45/159 (28%) modified protocols on scanner 1 and 38/150 (25%) modified protocols on scanner 2 had at least one inappropriate modification. Two of 98 (2%) inappropriate modifications were acquisition parameter modifications, while 96/98 (98%) inappropriate modifications were reconstruction parameter modifications (Fig. 6). The two inappropriate acquisition parameter modifications were both to the quality reference mAs, which is particularly important to flag as this parameter impacts both radiation dose and image quality. The most frequently affected parameter of the inappropriate modifications was the series description, which was inappropriately modified a total of 47 times. In our practice, the series description contains important information about the scan anatomy and region, the scanning technique, and the reconstruction method, and thus needs to be written in a specific manner. It is easy for a change to be made to the series description, making it very hard for a specific series to be tracked and kept consistent. Clinically, series description plays an important role in hanging protocols, the series of actions performed to arrange images for optimal softcopy viewing on a PACS or clinical image viewer system. As hanging protocols play a critical role in presenting specific types of studies in a consistent manner, and in reducing manual image ordering adjustments, correct series descriptors are very important for workflow efficiency, particularly for radiologists.

## PROTOCOL MONITORING SYSTEM, VERSION 2

Following our initial evaluation of the first version of the protocol monitoring system, we made several updates to the software. Most importantly, the pilot study highlighted several limitations of setting master protocols from baseline (Week 1) protocols on each scanner. First of all, this method assumed that Week 1 parameters were correct. If there was a mistake in the protocol at Week 1, it was not detected automatically because the master and scanner protocols each had the same mistake and were not flagged. Second, each scanner had its own set of master protocols and was only compared to itself, which did not allow for testing the consistency in protocols between scanners.

As an improvement, instead of using the Week 1 scanner protocols as the master, we switched to using our online protocols, which we call eProtocols. These eProtocols are maintained and edited in Microsoft Word, then published to PDF and posted on the web, where technologists use them as reference during patient exams. Using the eProtocols allows for one central set of master protocols for all scanners of the same model. This ensures that each scanner's protocols are consistent with the master and with each other. A MATLAB function is used to extract tables of protocol parameters using ActiveX controls in order to read the master protocols from Microsoft Word documents.

As another advancement in the workflow, we have worked with the scanner manufacturer to develop a method for automatically accessing the scanner protocols. This removes the need to manually export the protocols each time before running the software. This customized solution will be useful until the new DICOM standard is released and adopted by the vendors.

With the new version, we have also performed initial evaluations to investigate use of the software on body regions outside of abdomen and pelvis and on other scanner models (Siemens SOMATOM Force and Sensation 64 scanners). There are differences in scanner protocol files between different models, and these differences would be expected to be even greater between different vendors. However, the same principles and workflow still applied. It is expected that our software could be readily expanded to include multiple scanner makes and models.

A limitation of this work was that only the lead technologists reviewed the flagged scanner modifications and not the entire protocol review team. However, the decision on what the parameters should be came from the protocol review team, consisting of at least one radiologist, a tech, and a physicist. The lead techs were part of the protocol review team and were therefore aware of the appropriate protocol parameters that had been agreed on by the entire team. Furthermore, we did not investigate the reasons for the decisions between assigning the flagged protocol changes as appropriate or inappropriate. A detailed analysis of these decisions could help further optimize the protocol review process.

## V. CONCLUSIONS

This work has outlined our experience with the development and implementation of protocol monitoring software for the review of CT scanner protocols. The described protocol monitoring system minimizes the workload of protocol review as it automates the comparison of current scanner protocols against master protocols, which is an otherwise tedious manual process. Initial evaluation of this software demonstrated that CT protocol changes were frequent in our clinical practice, and that many of these changes were inappropriate modifications. This provides strong motivation for the use of an automated CT protocol quality control system that can ensure protocol accuracy and consistency.

## ACKNOWLEDGMENTS

We would like to thank Emily Sheedy and Michele Powell for their participation in and support of this work.

## COPYRIGHT

This work is licensed under a Creative Commons Attribution 3.0 Unported License.
